# Metabolic reprogramming shapes the immune microenvironment in pancreatic adenocarcinoma: prognostic implications and therapeutic targets

**DOI:** 10.3389/fimmu.2025.1555287

**Published:** 2025-03-21

**Authors:** Weihua Song, Yabin Yu, Siqi Wang, Zhengyi Cui, Qiusi Zhu, Wangrui Liu, Shiyin Wei, Jiachang Chi

**Affiliations:** ^1^ Department of Liver Surgery, Renji Hospital, School of Medicine, Shanghai Jiao Tong University, Shanghai, China; ^2^ Department of Hepatobiliary and Pancreatic Surgery, The Affiliated Huai’an No. 1 People’s Hospital of Nanjing Medical University, Huai’an, Jiangsu, China; ^3^ Department of Public Health, University of Texas Health Science Center at Houston, Houston, TX, United States; ^4^ College of Animal Science and Technology, Northeast Agricultural University, Haerbin, China; ^5^ Affiliated Hospital of Youjiang Medical University for Nationalities, Baise, China; ^6^ Key Laboratory of Tumor Molecular Pathology of Baise, Baise, China; ^7^ Department of Thoracic Surgery, Renji Hospital, School of Medicine, Shanghai Jiao Tong University, Shanghai, China

**Keywords:** pancreatic adenocarcinoma, multiple omics data, MPI score model, survival analysis, immune landscape

## Abstract

**Introduction:**

Pancreatic adenocarcinoma (PAAD) is characterized by a profoundly immunosuppressive tumor microenvironment (TME) that limits the efficacy of immunotherapy. Emerging evidence suggests that tumor-specific metabolic reprogramming may drive disease progression and shape the immune landscape in PAAD.

**Methods:**

We integrated multi-omics data from TCGA, GEO, and ICGC to identify key metabolism-related genes (MRGs) that influence immune cell infiltration, tumor progression, and patient survival. Based on nine pivotal MRGs (including ANLN, PKMYT1, and HMGA1), we developed and validated a novel metabolic-prognostic index (MPI). Functional enrichment analyses were conducted to elucidate the metabolic pathways associated with different MPI risk groups. *In vitro* experiments and drug sensitivity analyses were performed to confirm the oncogenic role of selected MRGs and to explore their therapeutic implications.

**Results:**

The MPI effectively stratified patients into high- and low-risk groups. High-MPI scores correlated with poor overall survival, elevated tumor mutation burden (TMB), and an immunosuppressive TME, evidenced by reduced CD8⁺ T-cell infiltration and increased expression of immune checkpoints (PD-L1, TGF-β). Functional enrichment revealed glycolysis and folate biosynthesis as dominant pathways in high-MPI groups, whereas fatty acid metabolism prevailed in low-MPI groups. Experimental validation underscored the role of ANLN in promoting epithelial-mesenchymal transition (EMT) and immune evasion via NF-κB signaling. ANLN knockdown significantly reduced glycolytic activity, tumor cell migration, and immune evasion. Drug sensitivity analyses indicated resistance to gemcitabine but sensitivity to afatinib in high-MPI patients. Although TIDE analysis predicted immune checkpoint inhibitor (ICI) resistance in high-MPI tumors, a subset of patients showed favorable responses to anti-PD-L1 therapy.

**Discussion:**

These findings provide a comprehensive framework for understanding how metabolic reprogramming shapes PAAD’s immunosuppressive TME and affects treatment outcomes. By accurately stratifying patients, the MPI serves as a promising tool to guide therapeutic decisions, including targeted therapy selection and immunotherapy prediction, ultimately offering potential for more personalized management of PAAD.

## Introduction

Pancreatic adenocarcinoma (PAAD) has a relatively low global incidence (1), ranking 10th among cancers worldwide (2). In 2020, PAAD accounted for an estimated 125,000 new cases and 26.1% of cancer-related deaths in China. Despite its low incidence, PAAD has a high mortality rate, ranking sixth or seventh globally among malignant tumors (3, 4), with 26.1% of PAAD-related deaths occurring in China (5). The prognosis of PAAD patients is closely related to their clinicopathological stage, which serves as a crucial basis for determining treatment strategies. Currently, PAAD treatment primarily relies on surgery, chemotherapy, and radiotherapy (6). In recent years, new therapeutic drugs, such as albumin-bound paclitaxel (7), Fluorouracil, as well as new treatment regimens like FOLFIRINOX (8), have shown significant efficacy in PAAD. However, a significant number of patients do not benefit from conventional sequential therapy, possibly due to the lack of effective biomarkers or predictive models. At present, the treatment of PAAD has entered the stage of genetic diagnosis and classification-based therapy (9). Studies have identified a subset of PAAD patients with specific gene mutations, and the use of targeted therapies or specialized drugs for these patients has shown significant improvements in prognosis (10).

The tumor microenvironment refers to the internal environment in which tumor cells arise and survive (11). It includes not only tumor cells themselves but also fibroblasts, immune and inflammatory cells, glial cells, and other surrounding cells (12), as well as interstitial cells, microvessels, and biomolecules infiltrating nearby areas (13). The characteristics of the tumor microenvironment mainly fall into three categories: hypoxia (14), chronic inflammation (15), and immunosuppression (16). Tumor metastasis depends on the tumor microenvironment, which promotes metastatic events and the formation of a distal metastatic microenvironment (17), facilitating tumor cell dissemination, implantation, and metastasis.

Unlike other solid tumors, the interstitial components of PAAD account for more than 80% of the tumor volume, wrapping around the tumor parenchyma to form a stromal barrier and contributing to PAAD progression (18). For example, pancreatic tumor cells activate Pancreatic Stellate Cells (PSC) by secreting fibroblast growth factor and TGF-β, recruiting PSC to the surrounding tumor cells (19). Activated PSC promotes tumor cell growth and proliferation by secreting growth factors and an abundant extracellular matrix (20). In addition, in PAAD, antitumor effector immune cells, such as CD4+, CD8+ effector T lymphocytes, and NK cells, are reduced or nonfunctional (21), while immunosuppressive cells, such as tumor-associated macrophages (TAMs), regulatory T lymphocytes (Tregs), and myeloid suppressor cells (MDSCs), are functionally active and proliferate in large numbers (22). Thus, a microenvironment conducive to PAAD immune escape can be created (23).

Due to the limited supply of oxygen and nutrients in the local tumor microenvironment (24), the occurrence and development of solid tumors are strongly influenced by metabolic stress, such as metabolic waste accumulation and pH changes (25). Tumor cells shape a unique tumor microenvironment (TME) to evade immune surveillance through metabolic adaptations that support their growth and metastasis, maximizing nutrient utilization to meet their energy and biosynthetic needs. Studies have found that oncogenic signals and tumor metabolites regulate cellular intrinsic metabolic remodeling and mediate metabolic communication between tumor cells and TME (26), contributing to the development of tumor intervention therapies (27). For example, in hypoxic tumor regions, tumor cells produce large amounts of lactate, which impedes T-cell activation and tumor immune surveillance (28). In addition, lactic acid promotes the differentiation and polarization of TAMs, induces an M2-like phenotype, and reduces antitumor immune activity (29). Metavert, an inhibitor of glycogen synthase kinase 3β and histone deacetylase, normalizes glucose metabolism in PAAD cells and converts M2-type TAMs into an anticancer M1 phenotype in a mouse model (30). These results highlight the importance of metabolic patterns in PAAD and their interaction with tumor microenvironment, warranting further exploration.

Tumor metabolic remodeling is the cornerstone of all malignant biological activities, including the initiation and progression of PAAD (31). Identifying metabolic intervention targets in the tumor microenvironment can inhibit the energy acquisition and biosynthesis of PAAD, thereby controlling disease progression. Tumor and immune cells share several metabolic pathways, and targeting tumor cell metabolism will inevitably affect some immune cell functions (32). In addition to providing prognostic insights, metabolic reprogramming models, such as the metabolic prognostic index, could guide treatment decisions in PAAD by identifying patients who may benefit from targeted therapies or immune interventions. By understanding how tumor metabolism influences the immune microenvironment, these models offer the potential to tailor therapeutic approaches for improved patient outcomes. This study aims to investigate metabolic reprogramming differences in PAAD using large-scale transcriptomic data to identify a balance between tumor inhibition and immune cell activity maintenance. It will provide new insights and directions for targeting the microenvironment and metabolic remodeling of PAAD.

## Methods

### Data download and preprocessing

The gene expression profile data of The Cancer Genome Atlas Program (TCGA) pan-cancer datasets were downloaded from the Xena Browser database (https://xenabrowser.netl), and batch-standardized profiles were corrected and log-transformed. The stemness index (mRNAsi) of The Cancer Genome Atlas Program (TCGA) pan-cancer data was also obtained from this database. Metabolism-related genes (MPI genes) were identified by downloading metabolic and protein interaction networks from published studies.

We get TCGA PAAD genome mutation data (WES), transcriptome data (RNA-Seq, *Z*-score standardized), and clinical information from the cBioPortal database (https://www.cbioportal.org/). WES includes single-nucleotide variants (SNVS) and small insertion-deletion mutations (indels). Overall survival data and clinical phenotype data of PAAD patients were also retrieved from the database. Factors such as history of chronic pancreatitis, diabetes, cancer location (pancreatic head or tail, etc.), grade, stage, drinking status, smoking status, gender, age, radiotherapy history, and others were considered (Table 1).

**Table 1:** TCGA PAAD sample clinical information summary table.

Number of patients (n)	176
Median age and range at diagnosis (years)	65 (35-88)
gender	
Female	80
Male	96
History of chronic pancreatitis	
Yes	13
No	127
Unknown	36
History of diabetes	
Yes	38
No	107
Unknown	31
Occurrence site	
Body of Pancreas	14
Head of Pancreas	137
Tail of Pancreas	14
Other	11
TNM staging	
T T1 (7) T2 (23) T3 (141) T4 (3) Unknown (2)	
N N0 (49) N1 (122) Unknown (5)	
M M0 (78) M1 (4) MX (94)	
AJCC Stage	
I (21) II (145) III (4) IV (4) Unknown (2)	
Grade	
G1 (31) G2 (93) G3(48) G4 (2) Unknown (2)	
Radiotherapy or not	
No	116
Yes	43
Unknown	17
Smoking	
No	64
Yes	79
Unknown	33
Drinking	
No	63
Yes	101
Unknown	12

The read count expression spectrum and Fragments Per Kilobase of transcript per Million mapped reads (FPKM) expression data for PAAD were downloaded from GDC (https://portal.gdc.cancer.gov/). An additional transcriptomic and clinical dataset for PAAD samples (PACA-AU) was obtained from the ICGC (https://licgc.otgl). Two PAAD datasets, GSE62452 and GSE21501, were downloaded from the GEO database (https://www.ncbi.nlm.nih.gov/gds). Clinical and transcriptomic data were used to validate the analysis. In addition, the R package IMvigor210CoreBiologies was utilized to extract clinical and transcriptomic data from 298 patients treated with the PD-L1 blocker atezolizumab.

### Pan-cancer analysis to identify metabolically altered genes specific to PAAD

MPI genes were obtained from published studies, and log-transformed, batch-standardized expression profile data were downloaded from the Xena Browser database. The Wilcoxon rank-sum test was used to identify differentially expressed gene sets, which were classified as PAAD-specific metabolism-related genes. PAAD samples were designated as the experimental group, while samples from other types served as the control group. The Benjamini and Hochberg method was used to adjust for multiple comparisons, and the fold change (FC) was calculated as the ratio of the median expression value in PAAD samples to the median expression value in samples from other tumor types. Selection criteria included a false discovery rate (FDR) ≤ 0.01 and an absolute |log2-transformed fold change (log2FC)| ≥ 3. The differentially expressed genes identified were classified as PAAD-specific metabolism-related genes (MIPros). Principal component analysis (PCA) was conducted using the R package Psych to validate these MIPros, and the first two principal components (PC1, PC2) were plotted.

The R package clusterProfiler was used to perform Gene Set Enrichment Analysis (GSEA) on the pan-cancer prognostic gene collection of MIPros in TCGA database. Gene expression levels in tumor samples were used as the independent variable, while the overall survival (OS) time was used as the dependent variable. The Cox proportional hazards model was applied to identify prognostic gene sets across pan-cancer data, with a selection threshold of *p* ≤ 0.05.

For the identified PAAD tumor-specific metabolism-related genes, PCA was performed using R-package psych to analyze these MIPros, and the first two principal components (PC1 and PC2) were plotted. A straight line was drawn according to PC1 = 3PC2, dividing PAAD samples into two groups, denoted as PCA subtypes C1 and C2. The log-rank test was used to assess the association between PCA subtypes and overall survival time, while the Wilcoxon rank-sum test was applied to examine the relationship between the PCA subtypes and Homologous Recombination Deficiency (HRD) score and TMB.

### Development and performance evaluation of the MPI score prognostic system

Based on the identified key module genes and differentially expressed genes of the PCA subtypes, a univariate Cox regression model was applied to identify prognostic factors with a significance threshold of *p* ≤ 0.05. Least Absolute Shrinkage and Selection Operator(LASSO)-logistic regression was then used to eliminate redundant factors and refine the selection of prognostic markers. The MPI score was calculated using the following formula, incorporating the proportional regression coefficient of risk from the Cox regression model and the expression levels of the selected prognostic factors, thereby constructing the prognostic risk score model:


$${\mathrm{MPI score}}_{i}\;=\;\sum_{j=1}^{n}C_{j}{\;}^{*}\exp_{ij}$$

This formula calculates the MPI score for the *i*th sample, where *C_j_* represents the regression coefficient of the *j*th prognostic factor in the Cox regression model, and exp*_ij_* denotes the expression level of the *j*th prognostic factor in the *i*th sample. The log-rank test was conducted to assess the correlation between the MPI score and patient survival time. The R package timeROC was used to generate the ROC curve and evaluate the prognostic performance of the risk model. Additionally, the correlation between the MPI score groups and clinical characteristics—including age, gender, tumor grade, TNM stage, and smoking/drinking status—was examined. The same method was applied to calculate the MPI score in the additional validation dataset. Samples were grouped based on the optimal threshold, and the prognostic performance of the high- and low-MPI score groups was assessed.

We used the Wilcoxon rank-sum test to analyze the association between PCA subtypes and MPI scores. The ESTIMATE method was applied to calculate the ImmuneScore of TCGA-PAAD samples. Spearman rank correlation was used to assess the correlation between MPI score and tumor purity, immune score, and stemness index. Additionally, the Wilcoxon rank-sum test was performed to compare differences in tumor purity, immune score, and stemness index between MPI score groups. The same test was used to examine differences in immune checkpoint (ICB) and Human Leukocyte Antigen (HLA) family expression levels between MPI score groups, while Spearman rank correlation was used to assess the linear correlation between MPI score and the expression levels of immune checkpoint and HLA family genes.

### Correlation analysis between MPI score groups and function

We downloaded the Kyoto Encyclopedia of Genes and Genomes (KEGG) pathway set and GO function from MSigDB(V7.4) (https://www.gsea-msigdb.org/gsea/msigdb/), including gene sets for Biological Process (BP), Cellular Component (CC), and Molecular Function (MF) secondary annotation classes. PAAD samples were grouped based on MPI score, and differential expression analysis was performed using the R package DESeq2 to obtain log2FC values related to MPI score grouping. These values were used as an ordered list of gene sets. The R package clusterProfiler was employed to conduct GSEA for the KEGG pathway and GO functional gene sets, respectively, and the R package EnhancedVolcano was used to generate a volcano map of the GSEA results.

We downloaded immune infiltration levels for TCGA-PAAD samples from the Tumor Immune Estimation Resource (TIMER) data resource (http://cistrome.dfci.harvard.edu/TIMER/). These immune infiltration levels were calculated using the TIMER, Cell-type Identification By Estimating Relative Subsets Of RNA Transcripts (CIBERSORT), A Gene Signature-Based Deconvolution Algorithm for Cell Types in Bulk Tissue (xCell), and Estimating the Proportion of Immune and Cancer cells (EPIC) methods. The Wilcoxon rank-sum test was used to assess the correlation between MPI score groups and immune infiltration levels.

Based on the MPI score, the top 30 genes with the highest mutation frequency were identified using the MAfTools package in R. An oncoplot was generated incorporating clinical information such as diabetes mellitus, history of chronic pancreatitis, cancer location (pancreatic head or tail, etc.), grade, stage, drinking status, smoking status, gender, age, and radiotherapy. The MAfTools package in R was also used to analyze the co-mutation patterns of the most frequently mutated gene to generate a lollipop plot of *TP53* mutations in high- and low-MPI score groups. Additionally, the WordCloud2 package in R was used to visualize gene distributions in high- and low-MPI score groups.

Based on the KEGG pathway set downloaded from the MSigDB (V7.4) database, KEGG pathways related to tumor metabolism were selected and categorized as metabolic pathways. The log2FC values associated with the MPI Sscore group were used as an ordered gene set list. The clusterProfiler package in R was used to perform GSEA on metabolic pathways, and a dot plot of the GSEA results was generated.

The immunotherapy Tumor Immune Dysfunction and Exclusion (TIDE) scores for the TCGA-PAAD dataset and validation sets PACA-AU, GSE62452, and GSE21501 were predicted using the TIDE service platform (http://tide.dfci.harvard.edu/). Wilcoxon rank-sum test and Spearman rank correlation were used to assess the association between the MPI score and TIDE across multiple datasets.

### Cell culture and incubation

The BxPC-3 cell lines were purchased from the American Type Culture Collection (ATCC, Gaithersburg, MD, USA). The cells were cultured in RPMI-1640 (Gibco, Grand Island, NY, USA) medium supplemented with 10% fetal bovine serum (FBS, Hycline, Life Sciences, Shanghai, China), 100 U/ml penicillin (Beyotime, Shanghai, China), and streptomycin (Gibco, Grand Island, NY, USA). All cells were maintained in a humidified incubator with 5% CO_2_ at 37°C (Thermo Scientific, Waltham, Massachusetts, USA).

BxPC-3 cells were transfected with double-stranded small-interfering RNA (siRNA) in a six-well plate using Lipofectamine 2000 reagent (RiboBio, Guangzhou, China), following the manufacturer’s protocol. The siRNAs targeting anillin (si-ANLN) and the control were obtained from Sangon Biotech (Shanghai, China). The BxPC-3 cells were harvested after at least 24 h of transfection and incubation for downstream experimental analysis. Single siRNA oligonucleotides targeting human ANLN (siRNA1: 5′-GCU ACA UUC UGU UCC CAA ATT-3′; siRNA2: 5′-CCA GAC CUC UGC UUU CAA ATT-3′) and a negative control siRNA were diluted in siRNA transfection medium (31985-070, Life Technologies, Waltham, Massachusetts, United States) and mixed with siRNA Transfection Reagent (13778150, Life Technologies) in RPMI-1640 medium, according to the manufacturer’s instructions.

BxPC-3 Cells were collected by scraping them into an SDS sample buffer supplemented with a mixture of protease inhibitors and PhosSTOP Phosphatase Inhibitors (Roche, Pleasanton, CA, USA). Western blotting was performed following standard procedures. The PVDF membranes were blocked and then incubated with primary antibodies against the following targets: ANLN (CL0303, Thermo Fisher Scientific, USA), E-cadherin (1:1,000, No. 3195, CST, Danvers, Massachusetts, USA), N-cadherin (1:1,000, No. 13116, CST), vimentin (1:1,000, No. 5741, CST), Snail (1:1,000, No. 3879, CST), TGF-β (1:1,000, No. 3711, CST), LDHA (1:1,000, PA5-27406, CST), MMP-2 (1:1,000, No. 40994, CST), MMP-9 (1:1,000, No. 3852, CST), P50 (1:1,000, No. 3035, CST), P65 (1:1,000, No. 4764, CST), VEGFA (1:1,000, No. 50661, CST), and GAPDH primary antibody (1:1,000, No. 5174, CST). A goat antirabbit IgG conjugated with HRP (1:3,000, ab205718, Abcam, Cambridge, United Kingdom) was used as the secondary antibody. Finally, the bands were visualized using the ECL-plus™ Western blotting chemiluminescence detection kit (BD Biosciences, Franklin Lakes, New Jersey, USA).

In the BxPC-3 cell invasion assay, 200 μl of serum-free medium containing 2 × 10^4^ transfected BxPC-3 cells was seeded into each hydrogel-coated upper chamber of Transwell inserts (hydrogel purchased from JetLife, China), while each lower chamber was filled with 800 μl of medium. After incubation for 12 h at 37°C, noninvading cells in the upper chamber were removed using cotton swabs. Subsequently, cells that had invaded through the Matrigel-coated filters were fixed with 4% paraformaldehyde for 15 min and stained with 0.1% crystal violet. The invaded cells on the underside of the filters were quantified using a microscope across five random fields, with the entire procedure being replicated three times. The migration assay followed the same procedure as the invasion assay, except that the upper chambers lacked the Matrigel coating (BD Biosciences).

Utilizing an EdU assay kit (Ribobio), the EdU incorporation assay was conducted according to the manufacturer’s guidelines. An overview of the procedure is as follows: initially, 2 × 10^4^ cells were plated per well on coverslips in 24-well plates and left to settle overnight. Subsequent to a 48-h incubation with MDL-800 (at 0 [DMSO vehicle only], 10, or 25 μM), the cells were exposed to an EdU-containing medium (50 μM final concentration) and incubated for an additional 2 h at 37°C. The cells were then fixed using 4% formaldehyde for 30 min, followed by permeabilization with 0.5% Triton X-100 for 10 min. An Apollo reaction cocktail was applied for 30 min at ambient temperature. To stain the DNA, Hoechst was used for 30 min, and the images were captured using a fluorescence microscope (Nikon Inverted Research Microscope ECLIPSE Ti, Tokyo, Japan) at ×20 magnification, with five random fields per well. Image analysis was performed using ImageJ software. The EdU incorporation rate was quantified as the percentage of EdU-positive cells relative to the total cell count in each visual field.

## Results

### The pan-cancer gene analysis to identify specific metabolic changes of PAAD

**Figure 1:**
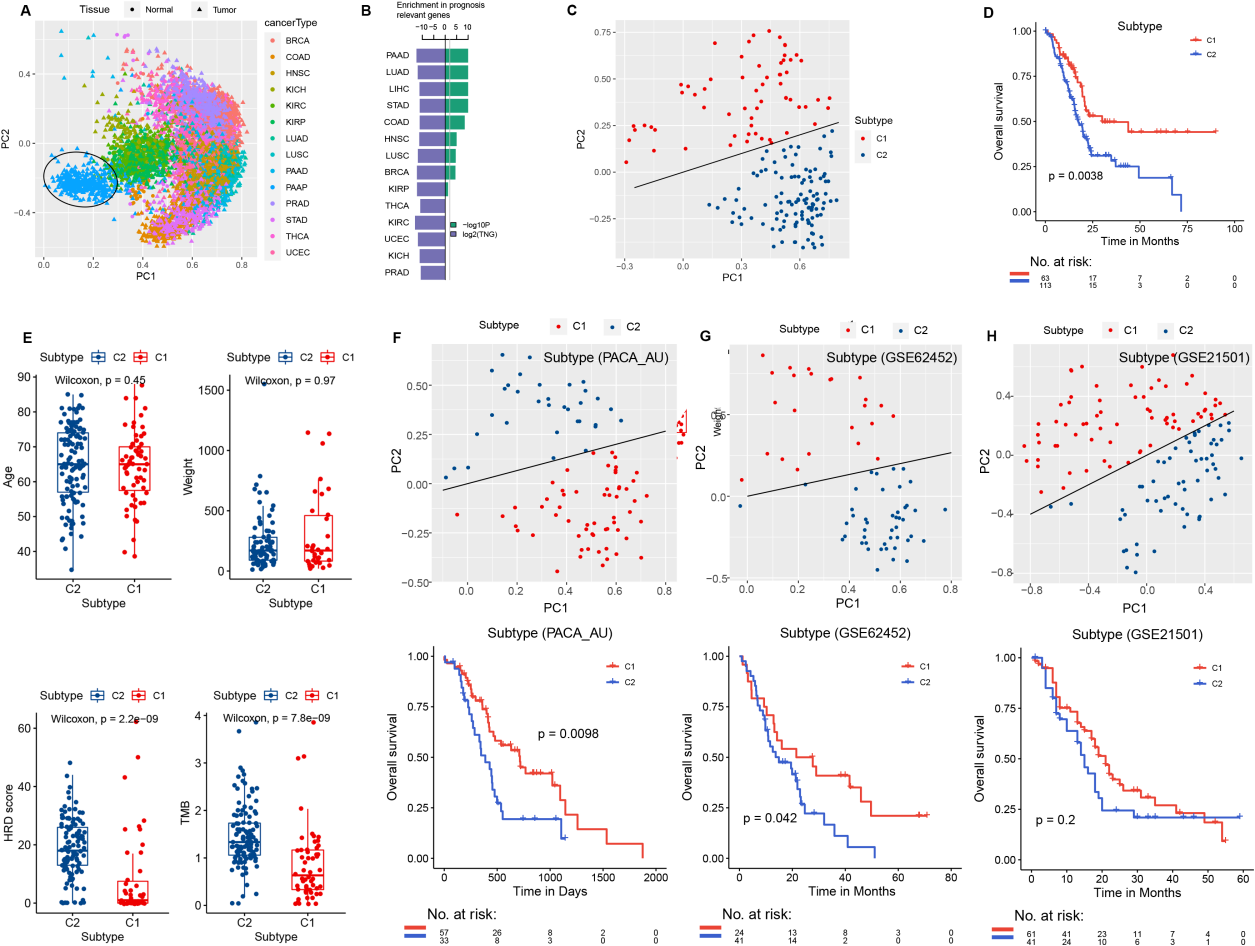
PCA and survival analysis. **(A)** Principal component analysis of the 14 tumor types in TCGA and the control normal tissues, with different colors indicating different tumor types. **(B)** Green: −log10P, the significance level of PAAD-specific metabolic genes in GSEA analysis of prognostic gene sets across 14 tumor types in TCGA. Blue: log2TNG, the prognosis-related gene set number (TNG, total number of the prognosis genes). **(C)** PCA of the expression levels of PAAD-specific metabolic genes (MIPros) in PAAD tumor tissues. **(D)** Survival analysis of PCA subtype groups. **(E)** Box plot of PCA subtypes and clinical features, including age, tumor weight, HRD score, and TMB. PCA analysis of PAAD-specific metabolic genes (MIPros) and survival of PCA subtypes in independent validation datasets: **(F)** PACA-AU, **(G)** GSE62452, and **(H)** GSE21501.

We obtained the interaction network between metabolism and genes from known studies, comprising 30,446 edges, and identified 4,112 MPI genes. Through pan-cancer analysis, 346 PAAD tumor-specific metabolism-related genes were identified (**Supplementary Table S1**). Based on the expression levels of these genes, principal component analysis was performed across 14 pan-cancer types, showing the first two principal components, PC1 and PC2. PAAD samples formed distinct clusters, clearly separating from other tissue types (**Figure 1A**). Next, Cox prognostic analysis was conducted across 14 cancer types, revealing a higher number of prognostic genes across all cancer types. GSEA enrichment analysis of the PAAD-specific metabolism-related gene sets showed that these genes exhibited the most significant enrichment in PAAD tumor types (**Figure 1B**).

Principal component analysis based on the expression levels of PAAD-specific metabolism-related genes identified two principal components, PC1 and PC2. Samples were classified into two PCA subtypes based on the equation PC1 = 3PC2, with sample sizes of 63 and 113, respectively (**Figure 1C**). Survival analysis revealed a significant difference between the two PCA subtypes (*p* = 0.0038, **Figure 1D**). In addition, significant differences in HRD score and TMB were observed between the two PCA subtypes (**Figure 1E**).

In addition, in the independent validation datasets PACA-AU, GSE62452, and GSE215O1, we classified samples into two groups based on PC1 = 3PC2 for PCA analysis. Significant differences in patient survival between the two PCA subtypes were observed in the PACA-AU and GSE62452 datasets (**Figures 1F, G**). In the GSE21501 dataset, although the survival difference between the two PCA subtypes did not reach the significance threshold, a trend toward divergence was observed (**Figure 1H**).

### Identification of MPI-related genes among PCA subtypes and validation of external data

**Figure 2:**
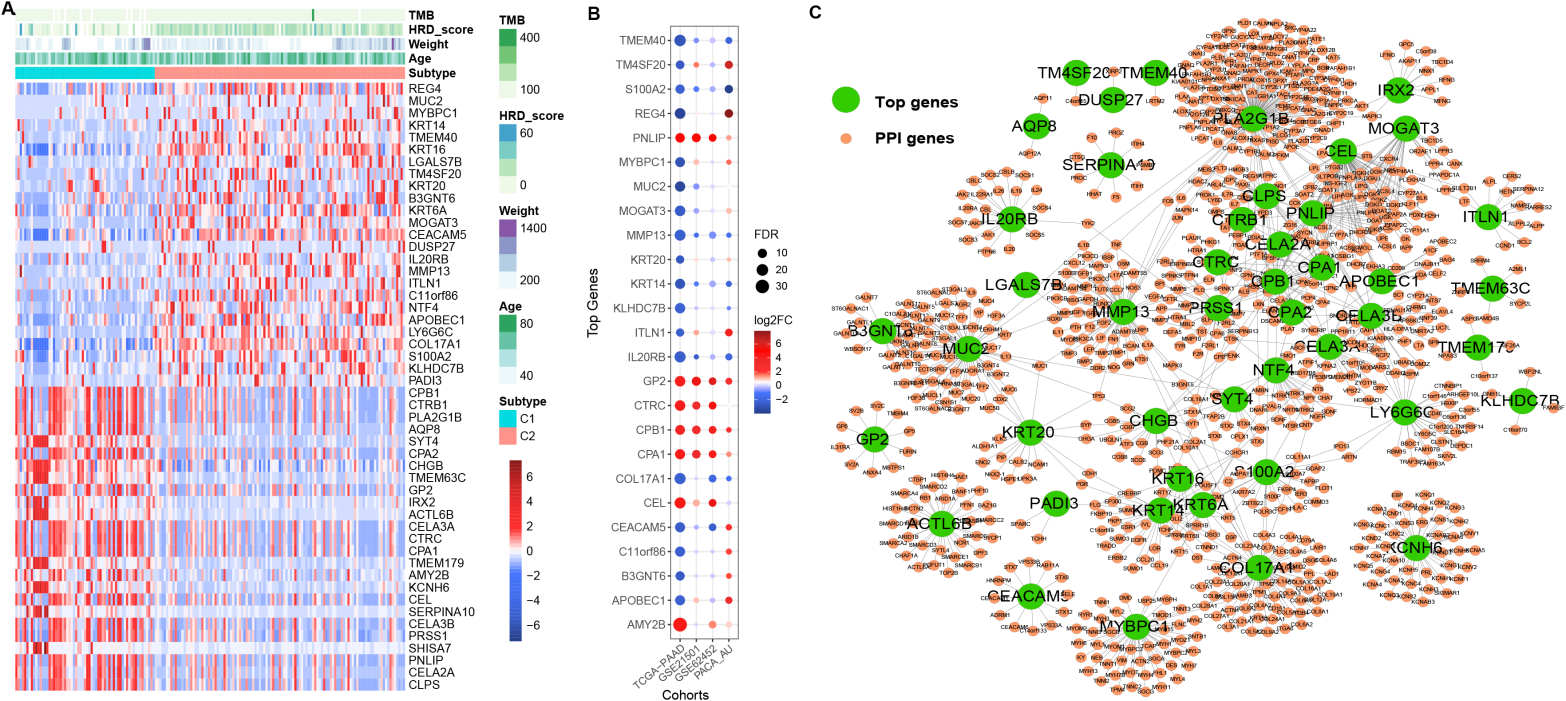
Differential expression and PPI. **(A)** Differentially expressed genes were identified based on PCA subtypes. Red indicates C1, and blue indicates C2, based on FC values. **(B)** Differential expression of the top 50 differentially expressed genes among PCA subtypes in TCGA-PAAD and independent validation datasets (PACA-AU, GSE62452, GSE21501). **(C)** Large dark green dots represent differentially expressed genes in PCA subtypes, while small orange dots indicate genes that are linked to them in the PPI network.

We performed differential expression analysis on the PCA subtypes of TCGA-PAAD and identified 2,165 differentially expressed genes (DEGs) (**Supplementary Table S2**). The expression levels of the top 50 genes in the two PCA subtypes were visualized, revealing significant differences between them (**Figure 2A**). In addition, most of these genes exhibited consistent differential expression patterns in the independent validation datasets (PACA-AU, GSE62452, GSE21501) (**Figure 2B**). Based on the STRING protein interaction network, we constructed the PPI network of the top 50 differentially expressed genes (**Figure 2C**; **Supplementary Table S3**).

### Identification of key modules related to PCA subtypes using WGCNA

**Figure 3:**
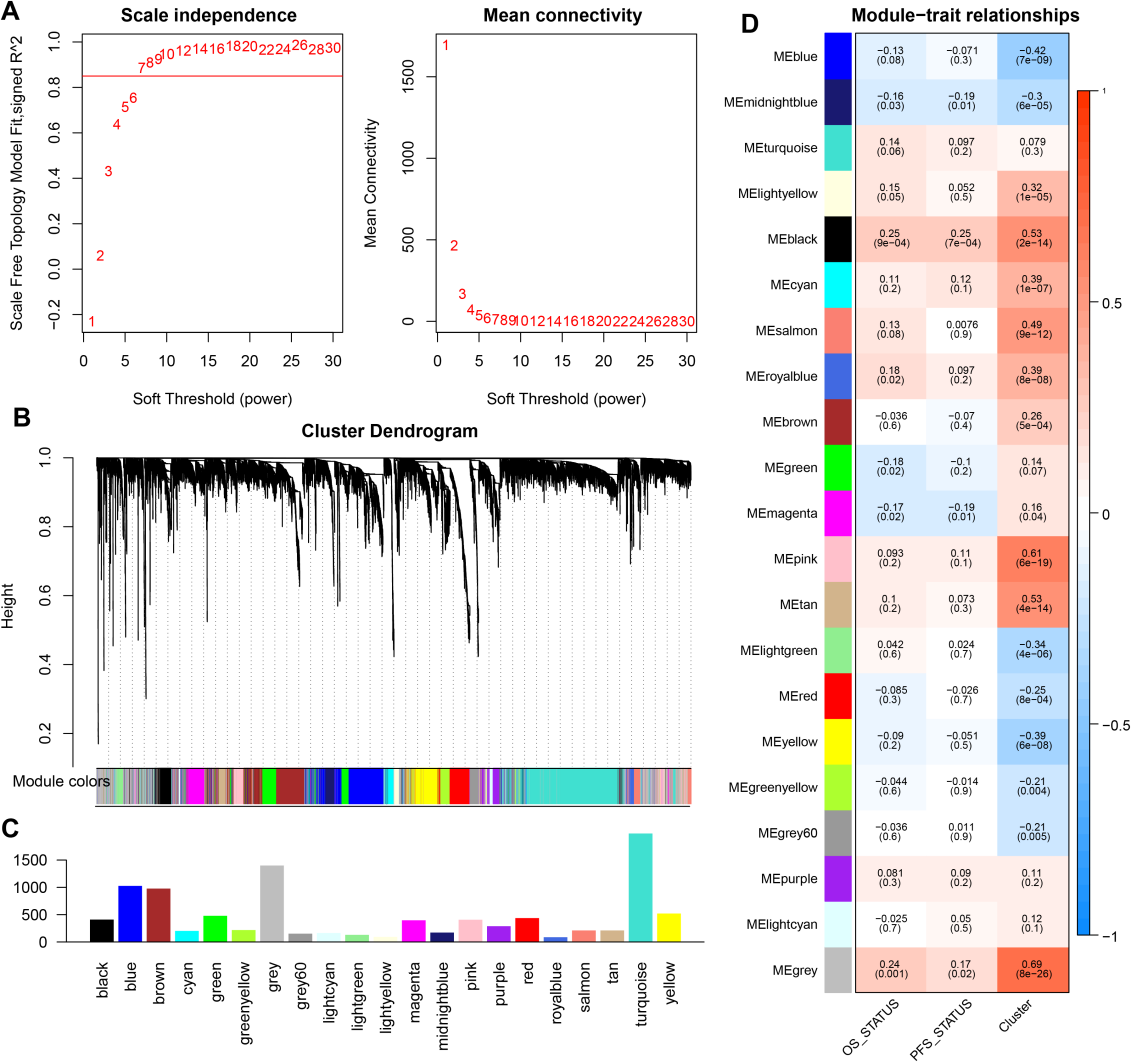
WGCNA analysis of gene expression levels in PAAD samples. **(A)** Distribution diagram of the soft threshold and average connectivity. The horizontal axis represents the soft threshold (power), while the vertical axis represents the evaluation parameter of the scale-free network. The higher the value, the more the network conforms to the scale-free feature. **(B)** The hierarchical clustering tree shows each module. Different colors represent genes grouped into different modules, while gray represents genes not classified into modules. **(C, D)** Association analysis of module genes with clinical phenotype data. Red indicates a greater positive correlation, while blue indicates a greater negative correlation.

We used the R package WGCNA to perform weighted correlation network analysis (WGCNA) on genes with the top 50% expression level fluctuation in the sample. The weighted gene network was constructed by calculating the Person correlation coefficient between gene pairs, and the soft threshold was determined through power calculation of the correlation values. Based on the distribution diagram of the soft threshold and average connectivity, we selected power = 7 (**Figure 3A**). A hierarchical clustering tree was then constructed using correlation coefficients between genes, where different gene modules are represented by distinct branches and colors. In this study, genes were classified into 20 modules, with the number of genes in each module shown in **Figures 3B, C**.

Based on the weighted correlation coefficients, genes were grouped into modules according to their expression patterns. When incorporating the PCA subtype as a clinical feature, correlation analysis revealed that the Black module exhibited a strong positive correlation with the PCA subtype (*R* ^2^ = 0.53, *p* = 2.0*E*−14) and was also correlated with OS status (*R* ^2^ = 0.25, *p* = 9.0*E*−04; **Figure 3D**). Therefore, the Black module was selected for downstream analysis in this study.

### Construction of MPI score model based on MPI-related and key module genes

**Figure 4:**
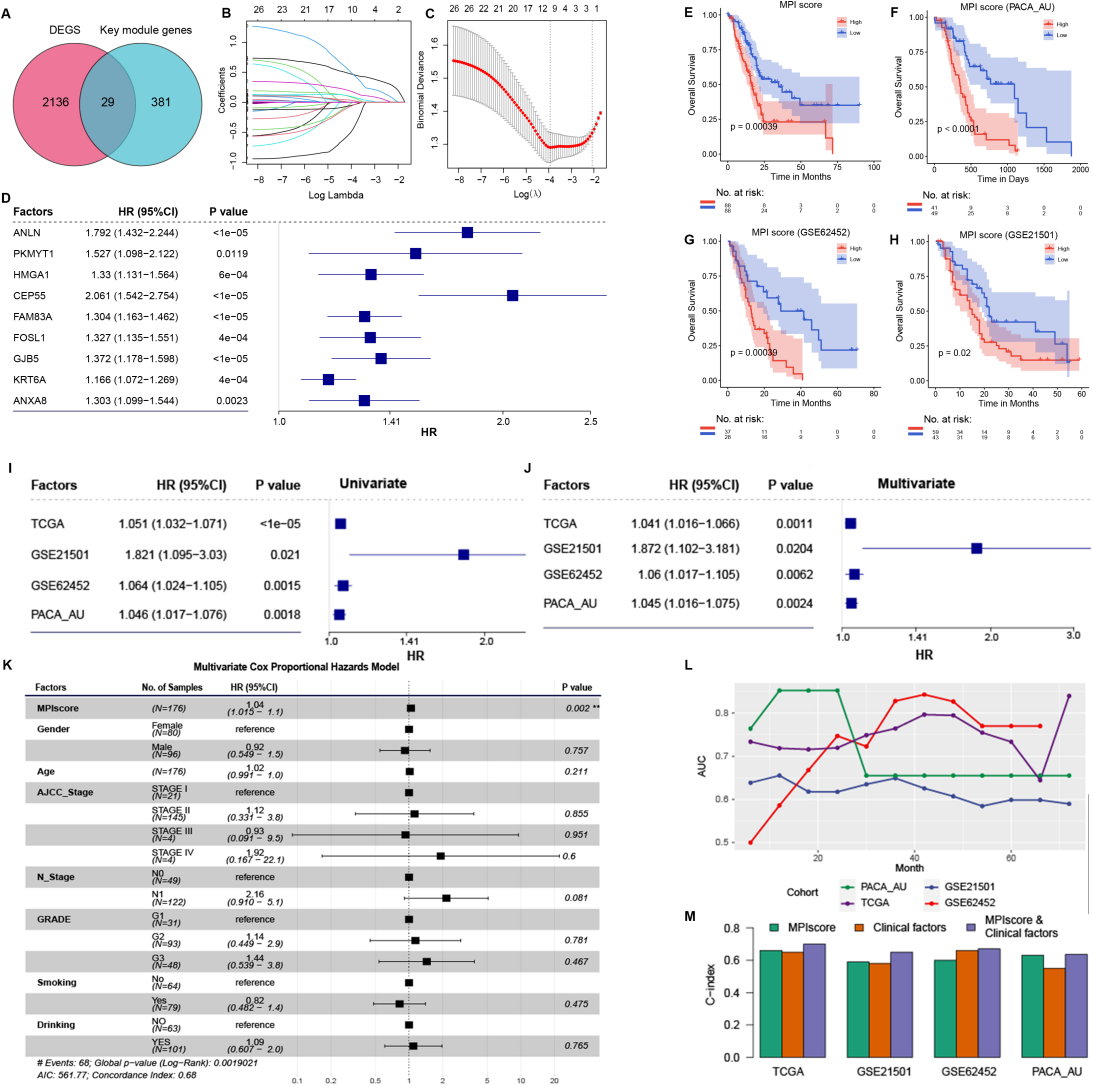
**(A)** Intersection of differentially expressed genes in PCA subtype and Black key module genes in WGCNA analysis. **(B)** The path diagram of LASSO regression illustrates how the coefficients of factors in the model change with varying *λ* values. **(C)** The *λ* value of the model was determined by 10-fold cross-validation, ultimately selecting nine prognostic factors. Patients were stratified into high- and low-MPI groups based on the median MPI score. **(D)** Cox proportional hazards model analysis assessed the prognostic efficacy of the nine prognostic factors. Hazard ratios (HRs) and corresponding confidence intervals indicate each factor’s independent impact on patient survival, with HRs greater than 1 signifying increased risk and HRs less than 1 suggesting a protective effect. This analysis highlights the robust prognostic value of the MPI score relative to other factors. **(E)**, Survival analysis of high- and low-MPI score groups in the MPI score model constructed using TCGA-PAAD data. **(F–H)** KM curves validate the prognostic efficacy of the MPI score model in independent datasets PACA-AU, GSE62452, and GSE21501. **(I)** Univariate Cox regression analysis in TCGA dataset and independent validation datasets. **(J)** Multivariate Cox regression analysis adjusting for clinical factors such as age, gender, and tumor grade. **(K)** Multivariate Cox regression analysis in TCGA dataset, adjusted for clinical factors including age, gender, and tumor grade. **(L)** ROC curve analysis of the MPI score model’s AUC values at different survival times in TCGA dataset and independent validation datasets. **(M)** C-index of different models in TCGA dataset and independent validation dataset. **p ≤ 0.01.

An intersection was taken between the genes contained in the identified key module Black and the differentially expressed genes of the PCA subtype (**Figure 4A**). Using a univariate Cox regression model, the prognostic factors were identified with a threshold of ≤ 0.5 (**Supplementary Table S4**), and redundant factors were removed through LASSO-logistic regression to further refine the selection of prognostic factors. The results indicated that the model achieved optimal efficiency when it included nine prognostic factors. Therefore, we selected these nine factors for subsequent analysis: ANLN, PKMYT1, HMGA1, CEP55, FAM83A, FOSLl, GJB5, KRT6A, and ANXA8 (**Figures 4B, C**).

Cox regression analysis confirmed that all nine prognostic factors were associated with increased risk factors (HR ≥ 1, *p* ≤ 0.05; **Figure 4C**). To evaluate the collective impact of these prognostic factors on patient survival, a prognostic score model was constructed based on their expression levels and Cox regression coefficients. The MPI score for each sample was then calculated (**Figure 4D**).

To assess the prognostic efficacy of the MPI score model, samples were divided into two groups based on the median MPI score. A significant difference in survival OS was observed between groups (**Figure 4E**). Additionally, independent validation datasets (PACA-AU, GSE62452, and GSE21501) were analyzed to confirm the prognostic accuracy of the MPI score model. KM survival curves demonstrated a significant difference in survival time between patients with high- and low-MPI scores (**Figures 4F–H**). These findings are consistent with results from the TCGA-PAAD dataset.

In addition, the prognostic efficacy of the MPI score model was assessed in TCGA dataset and independent validation datasets PACA-AU, GSE62452, and GSE21501. Univariate Cox regression analysis identified the MPI score as a significant risk factor for patient survival (**Figure 4I**). After adjusting for clinical factors such as age, gender, and tumor grade, multivariate Cox regression analysis confirmed that the MPI score remained an independent prognostic factor across these datasets (**Figure 4J**). Specifically, in the TCGA-PAAD dataset, multivariate Cox regression analysis demonstrated that the MPI score was an independent prognostic factor (HR = 1.04 [95% CI, 1.01–1.1]; *p* = 0.002; **Figure 4K**). Additionally, ROC curve analysis at different time points indicated strong prognostic performance of the model (**Figure 4L**). The C-index was also evaluated for the MPI score alone, clinical factors alone, and their combination. Notably, the combined MPI score and clinical factor model exhibited the highest C-index across datasets (**Figure 4M**).

### Correlation analysis between MPI score and clinical characteristics

**Figure 5:**
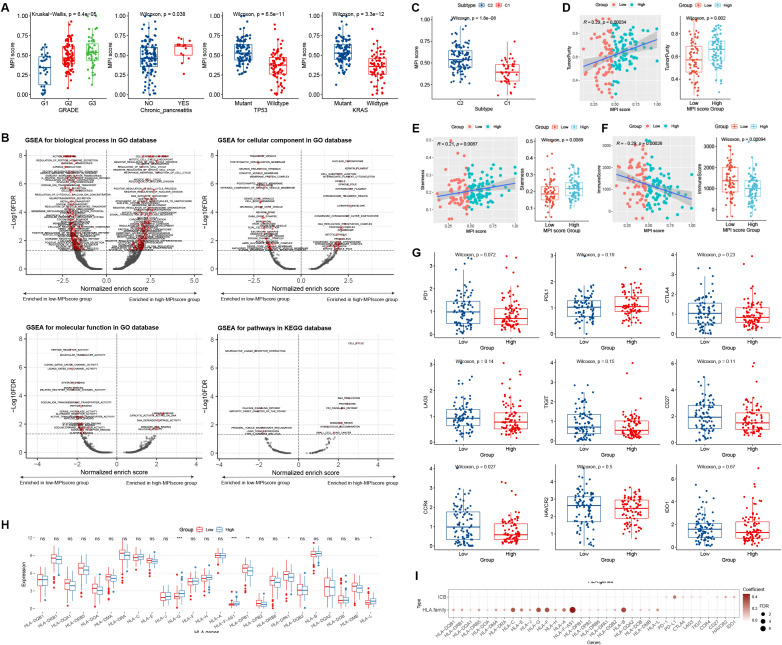
**(A)** Box plot distribution of MPI scores in TCGA dataset across different clinical feature groups. Statistical significance level was determined using the Wilcoxon rank-sum test for comparisons between two groups and the Kruskal–Wallis test for comparisons among multiple groups. **(B)** GSEA was used to analyze the functional enrichment of MPI score groups, including BP, CC, MF, and KEGG pathways. The horizontal axis represents the normalized enrich score (NES), where values less than 0 indicate enrichment in the low-MPI score group, while values greater than 0 indicate enrichment in the high-MPI score group. The dotted line indicates FDR = 0.05. **(C)** Box plot of MPI scores across PCA subtypes. **(D–F)** Correlation analyses between MPI scores and immune scores, dryness index, and tumor purity. The Wilcoxon rank-sum test was used to determine the significance level of box plots, while Spearman rank correlation was applied to scatter plots. **(G)** Box plot depicting MPI score groups and ICB factor expression levels. **(H)** Box plot showing MPI score groups and HLA family gene expression levels. ^*^ *p* ≤ 0.05; ^**^ *p* ≤ 0.01; ^***^ *p* ≤ 0.001; ns, not significant. **(I)** Correlation analysis between MPI scores and ICB factor and HLA family gene expression.

Statistical tests were conducted to examine the correlation between MPI scores and various clinical characteristics in TCGA-PAAD samples. No significant difference in MPI scores was observed across patients with different TNM stages, smoking/drinking status, gender, and age (**Supplementary Figure S1**). However, significant differences were found in relation to tumor grade, chronic pancreatic disease status, and TP53 and KRAS mutation status (**Figure 5A**).

We grouped PAAD samples based on the median MPI score. Using log2FC values of differentially expressed genes as an ordered gene set, GSEA analysis was performed for GO biological processes and KEGG pathways. The results were visualized using a volcano plot (**Figure 5B**). For example, the GO BP term MEIOTIC_CELL_CYCLE was significantly enriched in the high-MPI score group (**Figure 5B**), whereas the KEGG pathway NEUROACTIVE LIGAND_RECEPTOR_INTERACTION was significantly enriched in the low-MPI score group (**Figure 5B**).

We analyzed the distribution of MPI scores across different PCA subtypes and found that C2 patients had significantly higher MPI scores than C1 patients (*p* = 1.8*E*−08; **Figure 5C**). Additionally, the MPI score showed a significant positive correlation with both tumor purity and the stemness index, with notable differences observed between high- and low-MPI score groups (**Figures 5D, E**). Moreover, a significant negative correlation was found between MPI score and immune score, with significant differences between high- and low-MPI score groups (**Figure 5F**).

We explored the distribution of ICB and HLA family gene expression levels in groups with low- and high-MPI scores. For ICB expression levels, no significant differences were found between patients in the high- and low-MPI score groups (**Figure 5G**). However, certain HLA family genes exhibited significant differences in expression (**Figure 5H**). Additionally, linear correlation analysis showed a significant positive correlation between the MPI score and both ICB factors and HLA family gene expression levels (**Figure 5I**).

### Correlation between immune landscape and genomic mutations in MPI score groups

**Figure 6:**
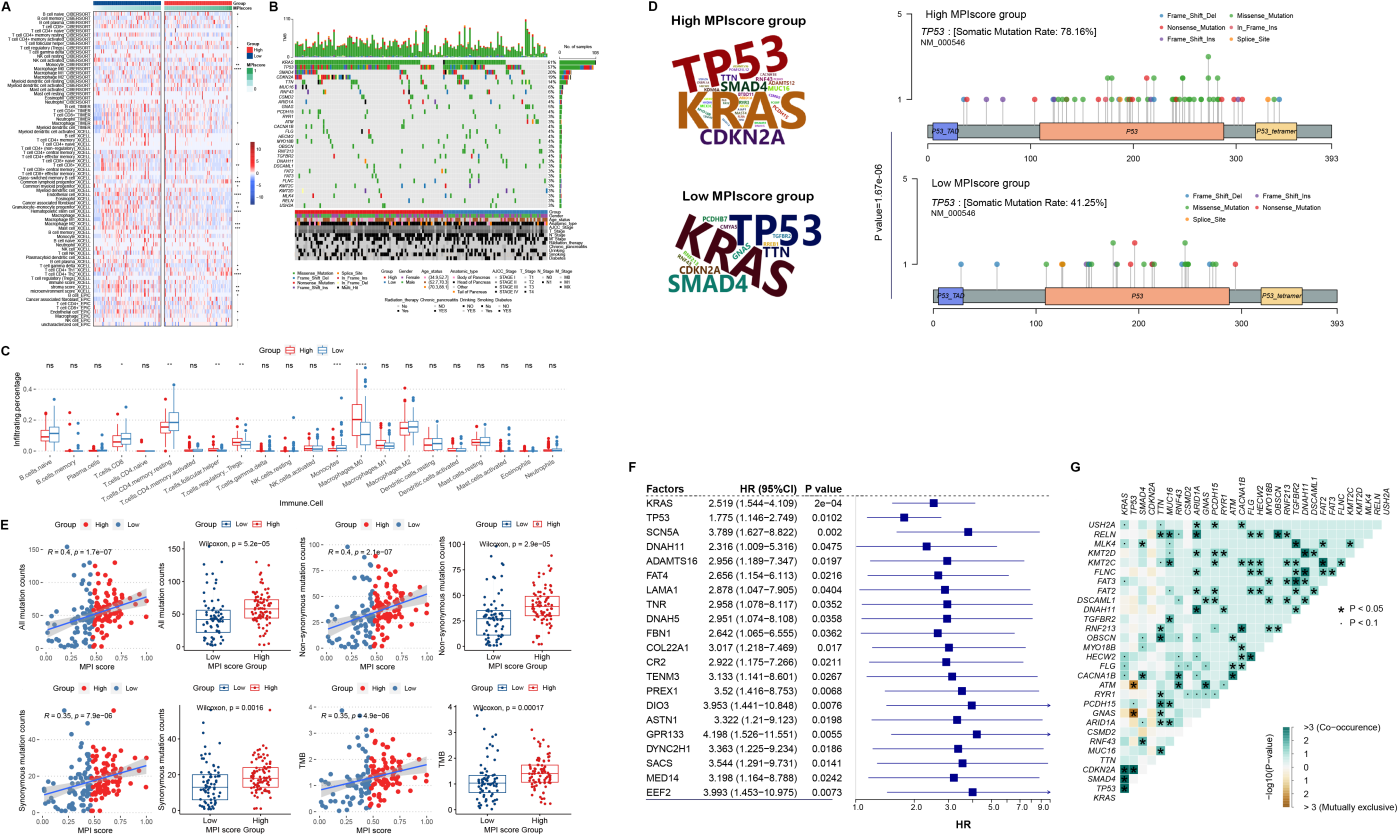
**(A)** Heat map of MPI score groups and immune cell infiltration levels, including TIMER, CIBERSORT, xCell, and EPIC. **(B)** Genomic mutation profiles of the high- and low-MPI score groups, displaying the top 30 mutated genes ranked by mutation frequency. **(C)** Box plot of MPI scores and CIBERSORT-based immune cell infiltration levels. Statistical significance levels were determined using the Wilcoxon rank-sum test. **(D)** Variation frequency of the top mutated genes in the high- and low-MPI score groups, with letter size representing mutation frequency. Mutation sites of the *TP53* gene in high- and low-MPI score groups. **(E)** Correlation between MPI scores and total mutation counts, nonsynonymous mutation counts, number of synonymous mutations, and tumor mutation burden (TMB). **(F)** Cox regression analysis of mutation status and overall survival (OS) time in genes with high mutation frequencies. **(G)** Co-mutation analysis of the top mutated genes in PAAD. In this plot, green indicates a statistically significant co-occurrence of mutations, suggesting potential cooperative effects in tumor progression or modulation of the immune microenvironment, whereas brown indicates mutual exclusivity, indicating alternative oncogenic pathways. ^*^ *p* < 0.05. **p ≤ 0.01; ***p ≤ 0.001; ns, not significant.

We downloaded different methods, including TIMER, CIBERSORT, xCell, and EPIC from the TIMER2.0 database, to calculate the immune infiltration levels in TCGA-PAAD samples and construct the immune landscape. Wilcoxon rank-sum test was applied to examine the correlation between MPI score groups and immune infiltration levels. Some immune cells exhibited significantly different levels of infiltration between high- and low-MPI score samples (**Figure 6A**). Furthermore, using FPKM expression profile data, we calculated infiltration scores for 22 immune cell types with the R package CIBERSORT, revealing significant differences between the high- and low-MPI score groups (**Figure 6C**).

In addition, we used the R package MAfTools to identify the top 30 genes with the highest mutation frequencies and examined their distribution in the high- and low-MPI score groups (**Figures 6B, D**). Fisher’s exact test was then applied to assess differences in mutation frequencies between the two groups, identifying 16 genes with significantly different variation frequencies in the high-MPI score group (**Supplementary Table S5**). For example, TP53 mutations were more prevalent in the low-MPI score group (*p* = 1.67*E*−06, **Figures 6B, D**). For all mutations, we found a significant positive correlation with the MPI score (**Figure 6E**), with the total number of mutations being significantly higher in the high-MPI score group (**Figure 6E**). Similar trends were observed for both nonsynonymous and synonymous mutations (**Figure 6E**). In addition, TMB was significantly elevated in the high-MPI score group (**Figure 6E**), further indicating a positive correlation with the MPI score (**Figure 6E**).

Based on the mutation frequencies ranked from highest to lowest, we selected the top 200 mutated genes. Univariate Cox regression analysis was then performed on mutation status, identifying 21 mutated genes associated with prognosis (**Figure 6F**). In addition, co-mutation analysis of the top 30 high-frequency mutated genes revealed significant co-mutation patterns in certain genes (**Figure 6G**).

GSEA analysis of tumor metabolism-related pathways showed consistent results across TCGA-PAAD dataset and validation sets RACA-AU and GSE62452 (**Supplementary Figure S2**). Notably, the Folate_biosynthesis and Glycosaminoglycan_biosynthesis_keratan_sulfate pathways were significantly enriched in high-MPI score samples across all three datasets, while the Fatty_acid_metabolism pathway was significantly enriched in the low-MPI score samples.

### Evaluation and analysis of drug response and immunotherapy effect of MPI score

**Figure 7:**
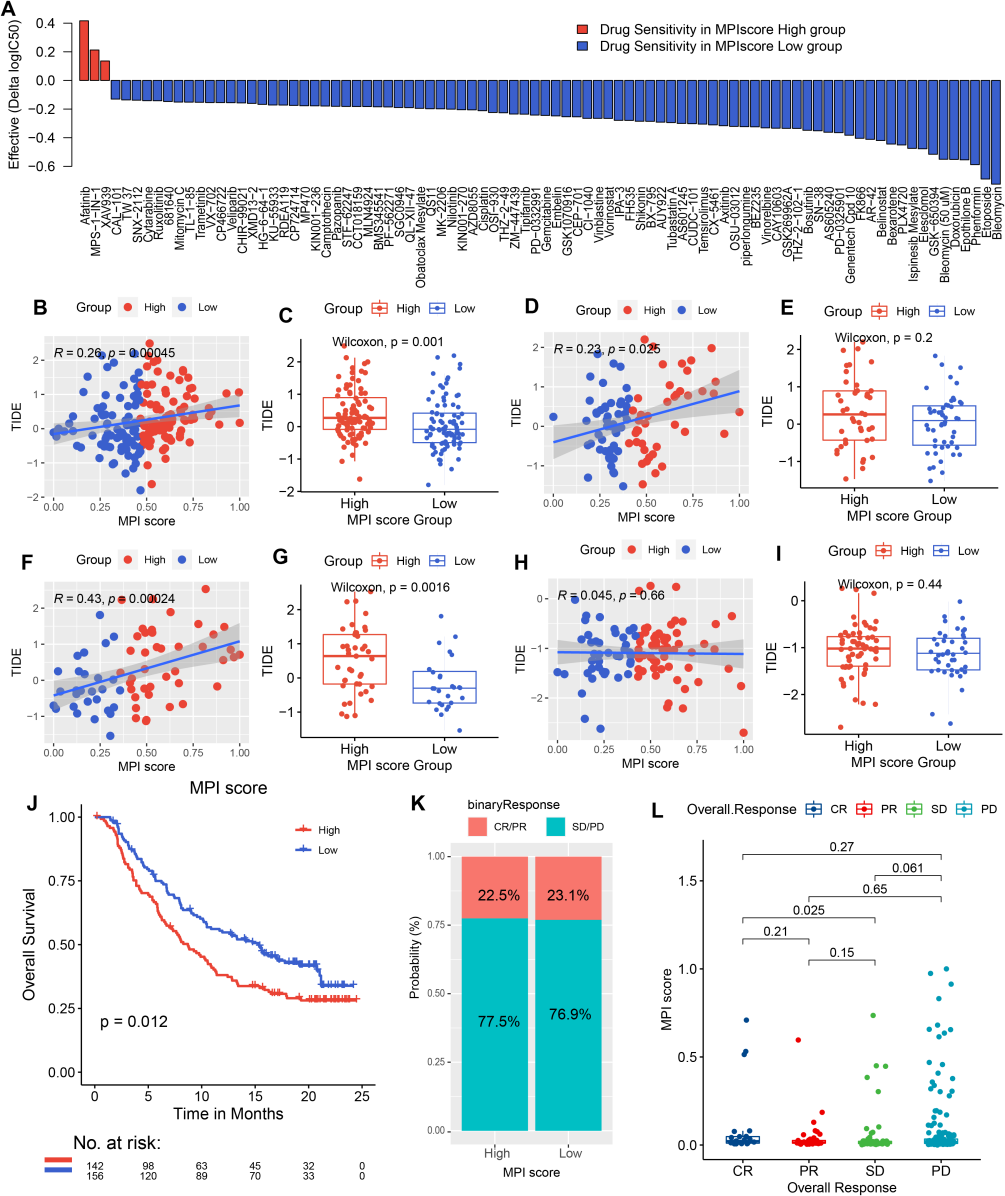
Drug sensitivity predicted by Ridge regression in the high- and low-MPI score groups of PAAD samples is shown in the bar chart. Red indicates that the drug is more electrically sensitive in the high-MPI score group, while blue indicates higher sensitivity in the low-MPI score group. The screening conditions were |Δlog2(IC_50_) ≥ 0.1, FDR ≤ 0.05. **(B**, **C)** Spearman correlation between MPI score and TIDE prediction score in TCGA samples, with box plot representation. **(D**, **E)** Spearman correlation between MPI score and TIDE prediction score, along with box plot representation in the PACA-AU dataset. **(F**, **G)** Spearman correlation between MPI score and TIDE prediction score, with box plot representation in the GSE62452 dataset. **(H**, **I)** Spearman correlation between MPI score and TIDE prediction score, with box plot representation in the GSE21501 dataset. **(J)** Survival analysis of immunotherapy-treated samples in the IMvigor 210 cohort, comparing high- and low-MPI score groups. **(K)** Proportion of immunotherapy response in high- and low-MPI score groups. **(L)** Correlation between response/nonresponse and MPI score was assessed using the Wilcoxon rank-sum test.

In addition, pharmacogenomic data from GDSC were downloaded, and ridge regression was applied to predict drug sensitivity in patients based on cell line expression data and drug response information (**Supplementary Table S6**). The correlation between high- and low-MPI score groups and drug response patterns was also explored (**Supplementary Table S7**). We found that for some anticancer drugs, patients with high-MPI scores were more sensitive to drug response, such as afatinib (**Figure 7A**). On the contrary, for etoposide, doxorubicin, and gemcitabine, patients with low-MPI scores were more sensitive to drug response (**Figure 7A**; **Supplementary Table S7**).

We predicted TIDE scores for immunotherapy in TCGA-PAAD dataset and validation sets PACA-AU, GSE62452, and GSE21501 (**Supplementary Tables S8**-**S11**). The correlation between the MPI score and the TIDE score was also analyzed, revealing a positive correlation in the three validation datasets (**Figures 7B, D, F**). For example, GSE62452 showed the strongest positive correlation (*R* ^2^ = 0.43, *p* = 2.4*E*−04, **Figure 7F**). Additionally, patients in the high-MPI score group had significantly higher TIDE scores across all three datasets (**Figures 7C, E, G**). However, in GSE21501, the correlation did not reach statistical significance (**Figures 7H, I**).

In order to explore whether the MPI score can be used as an immunotherapy response marker, the R package IMvigor210CoreBiologies was used to extract a set of transcriptomic and clinical data from patients treated with the PD-L1 blocker atezolizumab for validation analysis. A high-MPI score was found to be associated with better posttreatment outcomes (*p* = 0.012, **Figure 7J**). Additionally, the proportion of patients responding to atezolizumab (CR/PR) was similar between the high- and low-MPI score groups (high-MPI score: 22.5%; low-MPI score: 23.1%; **Figures 7K, I**).

### Tumor-promoting role of anillin *in vitro*

**Figure 8:**
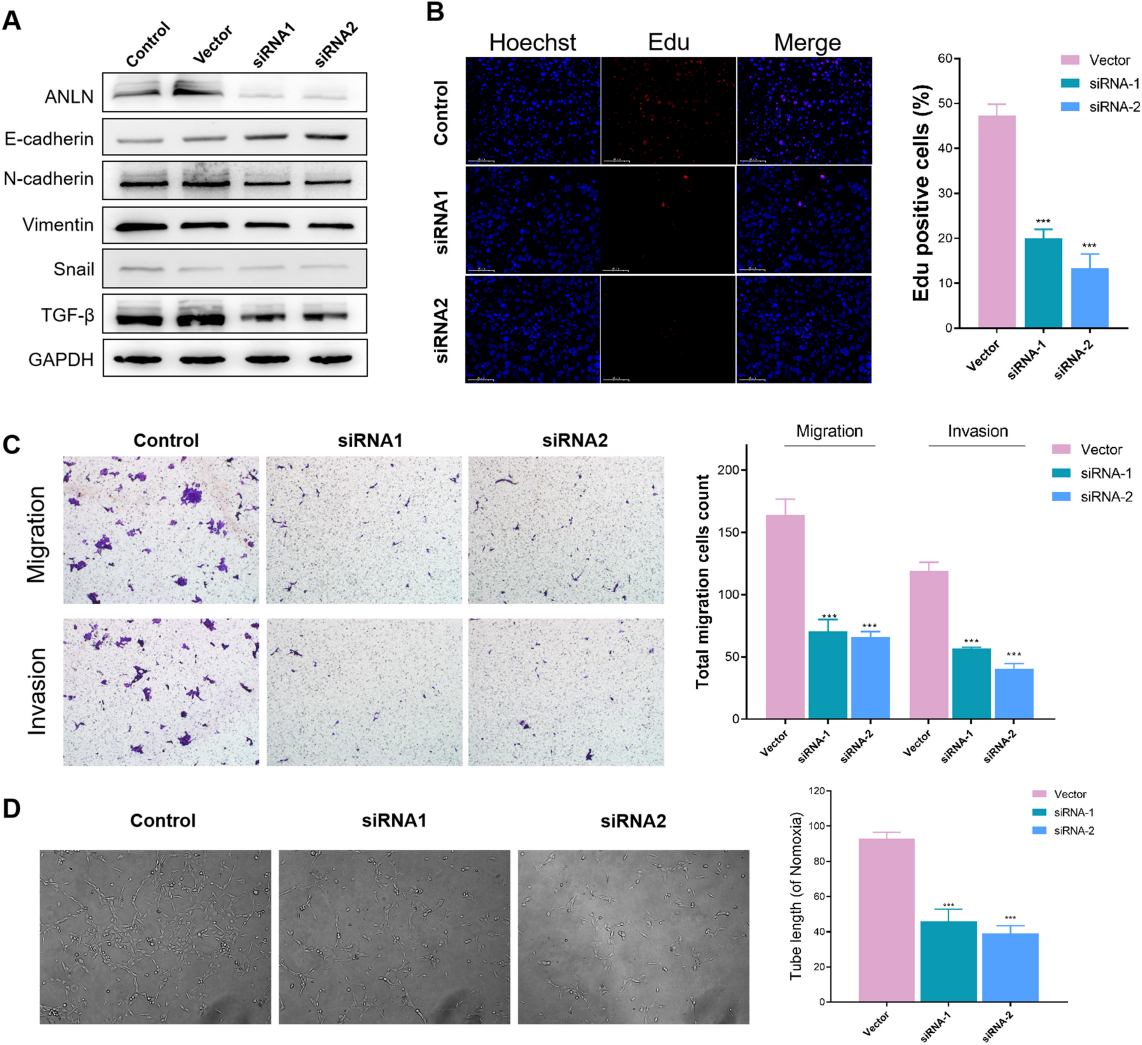
Anillin promotes epithelial-mesenchymal transition (EMT), proliferation, migration, invasion, and angiogenesis in BxPC-3 cells. **(A)** Western blot analysis confirming the knockdown efficiency of anillin (ANLN) in BxPC-3 cells transfected with siRNA1 and siRNA2. Knockdown of anillin resulted in increased E-cadherin levels and decreased expression of N-cadherin, Vimentin, Snail, and TGF-β, indicating inhibition of the EMT process. **(A)** EdU incorporation assay showing a significant reduction in proliferation in anillin-knockdown BxPC-3 cells compared to the control group (p < 0.001). **(C)** Transwell migration and invasion assays demonstrating a marked decrease in the migratory and invasive capabilities of anillin-knockdown BxPC-3 cells (p < 0.001). **(D)** Tube formation assay revealing a significant reduction in angiogenic potential following anillin knockdown (p < 0.001). All experiments were performed in triplicate, and statistical significance was determined using appropriate tests (p < 0.001). ***p < 0.001

Furthermore, we investigated the functional significance of anillin (ANLN) in BxPC-3 cells *in vitro*. We generated anillin knockdown (siRNA1 and siRNA2) human BxPC-3 cells and used Western blotting analysis to confirm knockdown efficiency (**Figure 8A**). The EMT process is widely recognized as a critical factor in cancer progression (33). Therefore, we assessed the levels of proteins associated with EMT (**Figure 8A**). The findings revealed an increase in E-cadherin and a decrease in N-cadherin, Vimentin, Snail, and TGF-β in anillin-knockdown BxPC-3 cells, suggesting that anillin may play an important role in facilitating the EMT process. To examine the effect of anillin on proliferation, we conducted the EdU pulse labeling, which revealed a significant reduction in anillin proliferative capacity in anillin-knockdown cells (*p* < 0.001) (**Figure 8B**). Next, Transwell assays were conducted to assess migratory and invasive capabilities (*p* < 0.001) (**Figure 8C**), showing significantly diminished migration and invasion in anillin knockdown BxPC-3 cells. Moreover, tube formation assays were performed to evaluate the impact of anillin on angiogenesis, which was remarkably reduced in anillin knockdown cells (*p* < 0.001) (**Figure 8D**). Collectively, these findings suggest that anillin plays a crucial role in promoting multiple aspects of tumor progression, including EMT, migration, invasion, and angiogenesis.

## Discussion

In recent years, the incidence of PAAD in China has been increasing year by year. The 5-year survival rate of PAAD is less than 9%, and 80% of patients are diagnosed at an unresectable stage. At present, PAAD frequently develops resistance to conventional treatments such as chemotherapy and radiotherapy, leading to rapid disease progression and poor prognosis. Although surgery remains the only potential curative treatment for PAAD, it is also associated with a high risk of local or distant recurrence (33, 34). Therefore, identifying new therapeutic targets has become an urgent priority.

Metabolic reprogramming is a hallmark of malignancy. In some cases, reprogrammed metabolic activity can be used to diagnose, monitor, and treat cancer. The characteristics of low oxygen and nutrient deficiency in the tumor microenvironment will lead to the establishment of metabolic competition between tumor cells and immune cells, and the accumulation of toxic metabolites will have a negative impact on the immune response (35–37). Additionally, the high metabolic activity and adaptability of tumor cells further change the metabolic characteristics of the tumor microenvironment, exerting metabolic pressure on infiltrating immune cells and promoting immune suppression and escape. Therefore, the interplay between the metabolic reprogramming of PAAD cells and tumor microenvironment is critical.

At present, specific tumor markers for PAAD have been studied. For example, pentraxin 3 (PTX3) is a sensitive and specific biomarker with an AUC of 91%, distinguishing PAAD from other cancers. PTX3 levels also fluctuate in response to drugs targeting cancer and stroma, and these changes can be easily measured in blood to monitor treatment effects (38, 39). However, whether PTX3 can serve as a biomarker for early detection in clinical practice remains to be determined. Therefore, we aim to identify multiple associations and markers for long-term prognosis and treatment prediction in PAAD.

In this study, pan-cancer analysis identified 346 PAAD-specific metabolic genes and patient samples were classified into two PCA subtypes using PCA analysis. These subtypes were correlated with patient survival time, HRD score, and TMB (40–42). Based on the differential expression analysis of the PCA subtypes and identified key module genes, we screened nine prognostic genes: ANLN, PKMYT1, HMGA1, CEP55, FAM83A, FOSL1, GJB5, KRT6A, and ANXA8. The MPI score prognostic evaluation system was constructed using these genes. At the protein expression level, we presented the heatmap distribution of the expression levels of the top 50 genes in the two PCA subtypes and plotted the PPI network of the top 50 differentially expressed genes. Unlike conventional prognostic models such as TNM staging or immune scores, the MPI score integrates metabolic reprogramming with immune landscape analysis, providing a more comprehensive and dynamic assessment of PAAD prognosis. While TNM staging remains essential for tumor classification, it does not account for the metabolic and immune complexities that significantly affect patient outcomes (43, 44). The MPI model, on the other hand, offers superior predictive value by incorporating tumor metabolism, immune infiltration, and drug sensitivity, thus guiding more personalized therapeutic strategies. Furthermore, the MPI score shows strong correlations with immune response markers and can predict immunotherapy outcomes, making it a promising tool for clinical decision-making.

GSEA analysis of the identified tumor metabolism-related pathways in TCGA and independent validation sets showed consistent enrichment of these pathways. Genomic analysis revealed a significant correlation between MPI score and PCA subtype, tumor purity, stemness index, and immune score. To detect *in vivo* drug sensitivity, we explored the correlation between the MPI score groups and drug response patterns. Notably, patients with higher MPI scores were found to be more responsive to certain cancer drugs (45–47). TIDE score predictions in TCGA database and three independent validation sets showed a positive correlation between the MPI score and TIDE score, suggesting that the MPI score may serve as a response marker for immunotherapy. To further validate this, we analyzed transcriptomic and clinical data from a group of patients treated with the PD-L1 blocker atezolizumab. The results showed that patients with high-MPI scores had better prognoses, confirming that the MPI score is a potential marker for PAAD immunotherapy response. The observed association between high-MPI scores and elevated PD-L1 expression suggests that patients in the high-MPI group may benefit from immune checkpoint inhibitor (ICI) therapies. Supporting this, our validation analysis demonstrated that PAAD patients with high-MPI scores who received PD-L1 blockers (e.g., atezolizumab) had improved prognoses. These findings imply that MPI could be a useful biomarker for guiding patient stratification for immunotherapy, although further clinical studies are warranted to confirm its predictive value.

Tumor metabolic remodeling is fundamental to the occurrence and progression of pancreatic cancer, serving as a cornerstone of its biological activities. Such metabolic remodeling by finding metabolic intervention targets to inhibit energy acquisition and biosynthesis has emerged as a new direction in pancreatic cancer research. Given its robust association with both metabolic reprogramming and the immune microenvironment, the MPI score could be integrated into clinical workflows to stratify PAAD patients. For instance, the MPI score may help identify patients who are more likely to benefit from targeted therapies or immunotherapies, thereby guiding personalized treatment decisions and optimizing therapeutic outcomes. The MPI score system presented in this study serves as an independent prognostic factor influencing patient survival and effectively reflects immune infiltration levels, anticancer drug sensitivity, and immunotherapy response in pancreatic cancer patients. It is a promising clinical marker for investigating the role and therapeutic impact of metabolic reprogramming in pancreatic cancer.

## Data Availability

The original contributions presented in the study are included in the article/**Supplementary Material**. Further inquiries can be directed to the corresponding author.

## Supplementary material

Supplementary Figure 2 GSEA analysis of tumor metabolism-related pathways. GSEA analysis of tumor metabolism-related pathways for high- and low-MPI score groups in TCGA-PAAD and independent validation datasets (PACA-AU, GSE62452, GSE21501).
