# Supplementary figures and images for: Metabolic reprogramming shapes the immune microenvironment in pancreatic adenocarcinoma: prognostic implications and therapeutic targets
Source: Front Immunol. 2025 Mar 21;16:1555287. doi: 10.3389/fimmu.2025.1555287 (PMC11968369; doi:10.3389/fimmu.2025.1555287)

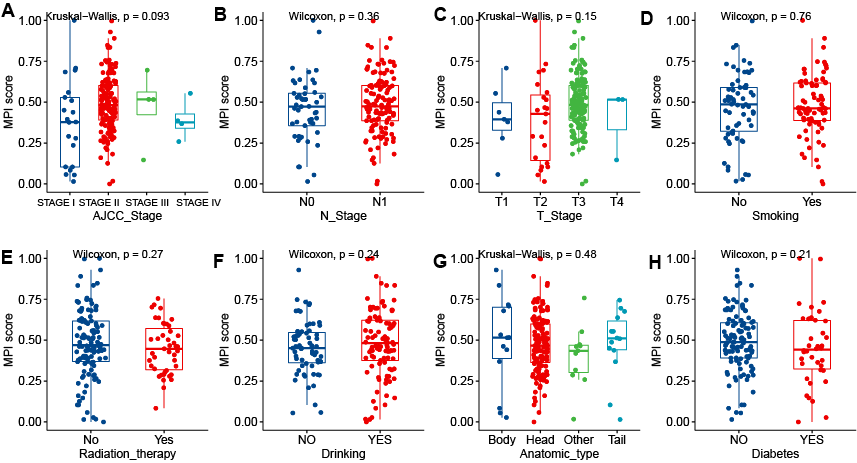

Supplement: Supplementary file 1 [file Image1.png]

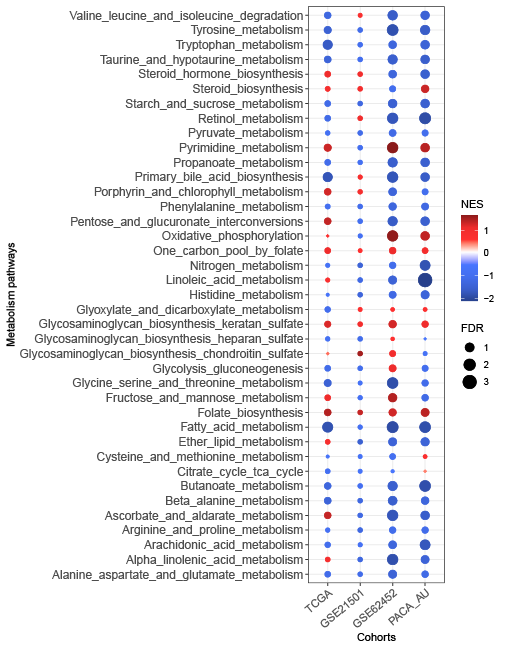

Supplement: Supplementary Figure 2 — GSEA analysis of tumor metabolism-related pathways. GSEA analysis of tumor metabolism-related pathways for high- and low-MPI score groups in TCGA-PAAD and independent validation datasets (PACA-AU, GSE62452, GSE21501). [file Image2.png]
